# Is PTSD-Phenotype Associated with HPA-Axis Sensitivity?: The Endocannabinoid System in Modulating Stress Response in Rats

**DOI:** 10.3390/ijms22126416

**Published:** 2021-06-15

**Authors:** Dor Danan, Doron Todder, Joseph Zohar, Hagit Cohen

**Affiliations:** 1 Anxiety and Stress Research Unit, Beer-Sheva Mental Health Center, Faculty of Health Sciences, Ben-Gurion University of the Negev, Ministry of Health, Beer-Sheva 8461144, Israel; doriandanan@gmail.com (D.D.); doron.todder1@PBSH.HEALTH.GOV.IL (D.T.); 2 Post-Trauma Center, Sheba Medical Center, Tel Aviv 5262000, Israel; joseph.zohar@sheba.health.gov.il

**Keywords:** posttraumatic stress disorder, hypothalamus-pituitary-adrenal axis, pulsatility, animal model, corticosterone, endocannabinoids, anandamide, 2-arachidonoyl glycerol

## Abstract

Endocannabinoids play a role in adaptation to stress and regulate the release of glucocorticoids in stressed and unstressed conditions. We recently found that basal corticosterone pulsatility may significantly impact the vulnerability for developing post-traumatic-stress-disorder (PTSD), suggesting that the endocannabinoid system may contribute to its development. To examine this, we exposed rats to predator scent stress (PSS). Behavioral reactions were recorded seven days post-PSS. Cerebrospinal fluid (CSF) was collected from anesthetized rats shortly after PSS exposure to determine the levels of 2-arachidonoyl glycerol (2-AG) and anandamide (AEA). To correlate between endocannabinoids and corticosterone levels, rats were placed in metabolic cages for urine collection. To assess the levels of endocannabinoids in specific brain regions, rats’ brains were harvested one day after behavioral analysis for staining and fluorescence quantification. Moreover, 2-AG was elevated in the CSF of PTSD-phenotype rats as compared with other groups and was inversely correlated with corticosterone urinary secretion. Eight days post-PSS exposure, hippocampal and hypothalamic 2-AG levels and hippocampal AEA levels were significantly more reduced in the PTSD-phenotype group compared to other groups. We posit that maladaptation to stress, which is propagated by an abnormal activation of endocannabinoids, mediates the subsequent stress-induced behavioral disruption, which, later, reduces neuronal the expression of endocannabinoids, contributing to PTSD symptomology.

## 1. Introduction

Hypothalamus-pituitary-adrenal (HPA) negative feedback inhibition occurs on a rapid time scale and is known to act via non-genomic routes. These non-genomic routes are thought to be mediated through endocannabinoid release [1,2,3,4,5]. The endocannabinoid system (ECS) is a neuromodulatory system that plays important roles in central nervous system development, synaptic plasticity, and the response to stress, the best-studied endogenous cannabinoids are 2-arachidonoyl glycerol (2-AG) and anandamide (AEA) [6]. AEA is thought of as a basal regulating molecule of the ECS. In the context of the hypothalamic-pituitary-adrenal (HPA) axis, it was found to be correlated with chronic/basal levels of glucocorticoids (GC) [7,8], whereas 2-AG is thought to induce a rapid and robust response upon stress, and the rapid increase of 2-AG is induced upon acute stress [9], mediating the fast-feedback inhibition of the HPA axis [5,10]. The primary mechanism mediating this rapid increase of 2-AG upon acute stress is the release of GC [9]. This GC-stress-induced increase in 2-AG that modulates PVN neurons is important for several aspects of the stress response; mainly, it contributes to negative feedback and the termination of the stress response, in addition to the development of habituation and adaptation under conditions of repeated exposure to stress in animals [11,12,13] and in humans [14,15]. In the amygdala, the acute stress-induced increase of 2-AG is positively correlated with amygdala corticosterone levels [16]. As expected, pharmacological augmentation of 2-AG signaling reduces the acute stress-induced peak of corticosterone, but it also prolongs the recovery of the HPA-axis response to stress [17]. A recent study in mice has demonstrated that stress-resilience is associated with increased stress-induced 2-AG-mediated synaptic suppression at ventral hippocampal-amygdala glutamatergic synapses, and that amygdala-specific 2-AG depletion impairs successful adaptation to repeated stress, suggesting that 2-AG signaling mechanisms modulate susceptibility and promote resilience to adverse effects of acute traumatic stress and facilitate adaptation to repeated stress exposure [18].

The contribution of 2-AG-CB_1_ signaling to the extinction of conditioned fear responses has led to extensive research into the pharmacological augmentation of 2-AG for its therapeutic potential in the treatment of anxiety disorders, including PTSD with conflicting results [19,20,21,22,23,24,25,26,27,28]. PTSD is brought on by exposure to a traumatic or stressful event [29] and is known to be associated with the HPA axis [30,31,32], and primarily with inadequate GC release following stress, which delays recovery by acutely disrupting biological homeostasis and, as in the case of endocannabinoids, interferes with the processing or interpretation of stressful information [33].

Recently, we have found that reduced basal, pre-trauma, corticosterone pulse amplitude is a pre-existing susceptibility factor for a blunted corticosterone stress response and the development of the subsequent PTSD-phenotype [34]. In theory, this dysregulation can be achieved through several pathways, among them is a disruption in the endocannabinoid system. This has led our team to investigate the rule endocannabinoids play in the development and aftermath of PTSD.

## 2. Results

**Figure 1:**
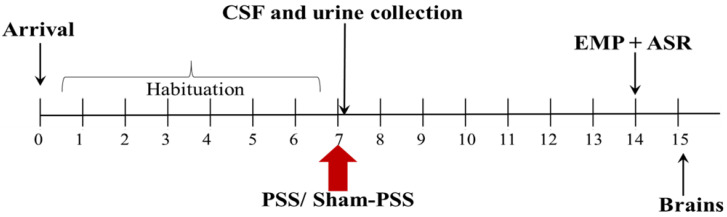
Timeline of the experiment after a 7-day habitation period; rats were exposed to predator scent stress (PSS)/sham PSS. Cerebrospinal fluid (CSF) samples were collected 60–120 min after PSS for evaluated levels of 2-arachidonoyl glycerol (2-AG) and anandamide (AEA). Urine corticosterone levels 60–120 min following exposure were also monitored. Behavioral assessments were conducted 7 days post-exposure to PSS, first in the Elevated Plus Maze (EPM) paradigm, and, 1 h later, in the Acoustic startle response (ASR) paradigm; on the following day, rats were executed and their brains were harvested for analysis.

During a stressful event, the HPA axis is activated by secreting its end product GC (cortisol in humans and corticosterone in the rat GC secretion rapidly suppresses stress-induced HPA activation via an endocannabinoid-dependent mechanism) [35,36]. To investigate whether the dysregulation of the stress-response, resulting in PTSD—phenotype rats, is mediated by the endocannabinoid system, we housed Sprague-Dawley rats for a 7-day habitation period, and rats were exposed to PSS/sham PSS (cat urine-soaked litter). CSF samples were collected 60–120 min after PSS, using a stereotactic apparatus, for evaluating levels of 2-AG and AEA. Urine corticosterone levels 60–120 min following exposure were also monitored. On the 7th day post-PSS exposure, we conducted behavioral testing based on the Cut-Off Behavioral Criteria (CBC) model [37,38,39,40]. These included elevated plus-maze (EPM) and acoustic startle response (ASR) test. Individual rats were classified as having an “extreme”, a “partial”, or a “minimal” behavioral response (EBR, PBR and MBR respectively), reflecting the magnitude of the response, according to preset cut-off criteria for behavioral response patterns; on the following day, rats were executed, and their brains were harvested for analysis (Figure 1).

**Figure 2:**
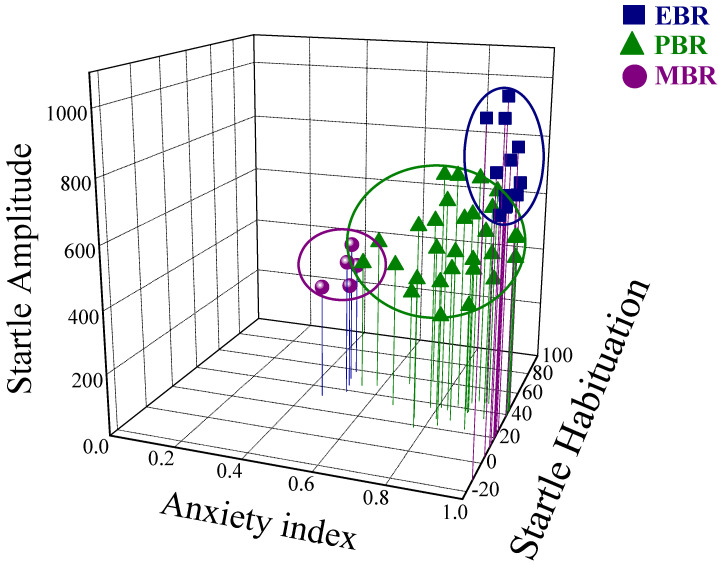
Behavioral responses Effects of the predator scent stress paradigm on overall anxiety-like behavior and startle response. Three-dimensional parameters: The *X*-axis represents time spent in the open arms (min), the *Y*-axis represents acoustic startle amplitude, the *Z*-axis represents startle habituation. Squares represent the exposed group that exhibited an extreme behavioral disruption (EBR) (PTSD phenotype) on the elevated plus-maze, and a pattern of exaggerated startle responses with significantly reduced habituation, 7 days after exposure. Triangles represent the exposed group that exhibited partial behavioral response patterns (PBR). Circles represent the exposed group that exhibited minimal behavioral disruption (MBR). The stress response was not homogeneous, and several subgroups were identifiable in the population.

Within the PSS-exposed population, 3 subgroups were identifiable (Figure 2). Assessment of the behavioral responses to PSS showed approximately 26% of the exposed animals (*n* = 12/46) fulfilled criteria for PTSD phenotype (EBR); 11% (*n* = 5/46) for MBR; and 63% for PBR (*n* = 29/46). For a better comparison of behaviors, 16 controls were added and exposed to sham-PSS, i.e., no cat urine, bringing a total of *n* to 62.

### 2.1. Shortly after PSS Exposure-CSF Endocannabinoids and Urine Corticosterone Concentrations

**Figure 3:**
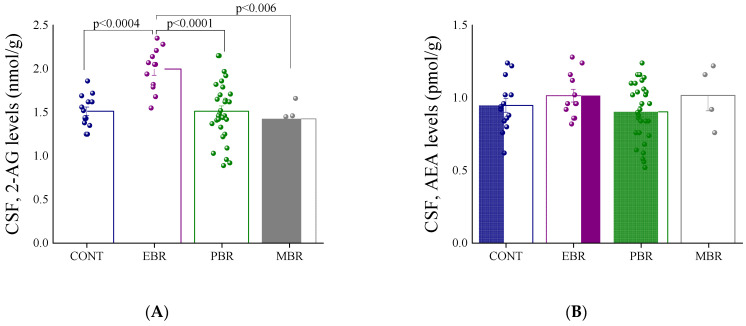
Cerebrospinal fluid (CSF) endocannabinoid levels shortly after predator scent stress (PSS) exposure (**A**) Graph showing 2-arachidonoyl glycerol (2-AG) levels in the CSF 60–120 min following PSS, in controls and in the different cut-off behavioral criteria (CBC) groups. Significant differences are shown on the graph. (**B**) Graph showing anandamide (AEA) levels in the CSF 60–120 min following PSS, in controls and in the different CBC groups. C.

To determine endocannabinoid levels 60–120 min following PSS, we examined the levels 2-AG and AEA in the CSF (Figure 3). In 2-AG levels, there were significant differences among the groups (one-way ANOVA: F(3,56) = 9.7, *p* < 0.0001) (Figure 3A). Bonferroni post hoc tests confirmed significantly higher 2-AG levels in the CNS in the EBR group than in the unexposed control (*p* < 0.0004), MBR (*p* < 0.006), and PBR (*p* < 0.0001) rats. No significant differences were found in AEA among the groups (Figure 3B). These findings indicate that a PSS-induced increase in CSF endocannabinoid levels was specific for 2-AG, since AEA levels were not affected by the PSS. Furthermore, the more robust 2-AG response seen in the EBR group indicates a stronger response of feedback inhibition mediated by 2-AG in the group that would later develop PTSD-phenotype.

**Figure 4:**
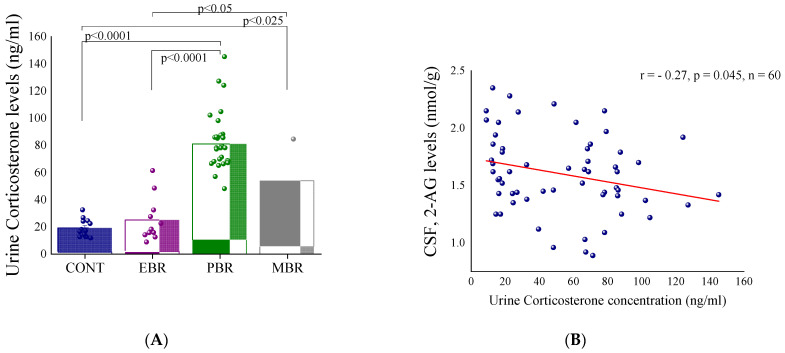
Urine corticosterone concentrations shortly after predator scent stress (PSS) exposure. (**A**) Urine corticosterone concentrations 60–90 min following PSS in controls and in the different cut-off behavioral criteria (CBC) groups. Significant differences are shown on the graph. (**B**) Correlation between 2-arachidonoyl glycerol (2-AG) levels in the cerebrospinal fluid (CSF) and urine corticosterone shortly after PSS. Significant values are shown in the graphs.

We next evaluated the urine corticosterone concentrations 60–90 min following PSS. There were significant differences among the groups (one-way ANOVA: F(3,56) = 40.4, *p* < 0.0001) (Figure 4A). All groups responded to PSS-exposure with significant elevations in corticosterone levels, although the response of the EBR group was significantly blunted compared to the MBR and PBR groups. Bonferroni tests confirmed that both the control and the EBR groups exhibited significantly lower corticosterone concentrations compared to the PBR (*p* < 0.0001 for both groups) and MBR group (*p* < 0.025 and *p* < 0.05, respectively). No significant differences were found between the control group and the EBR group.

We conducted further regression analyses to gain additional understanding of the relationship between CSF endocannabinoids levels and the urine corticosterone concentrations, irrespective of the CBC classification. Pearson’s correlation analysis revealed that there was a weak correlation between 2-AG levels in the CSF and urine corticosterone concentrations (r = −0.27; *p* < 0.045) (Figure 4B). CSF AEA levels did not correlate with corticosterone concentrations.

### 2.2. 8 Days Post-PSS Exposure-Brain Endocannabinoid Levels

**Figure 5:**
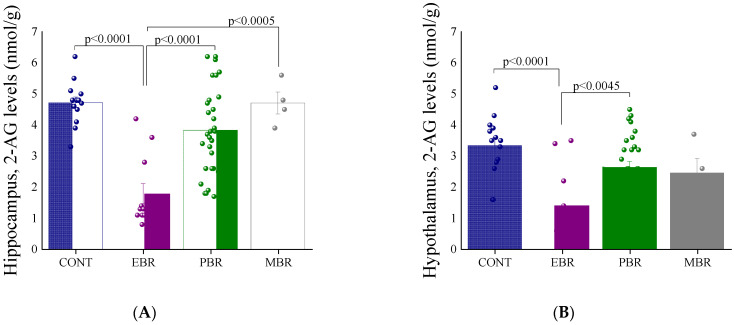

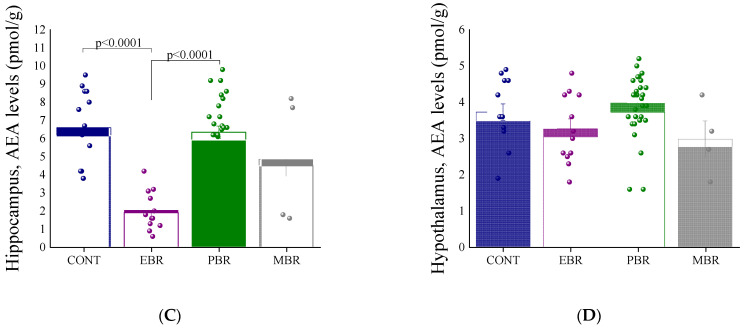
Brain endocannabinoid levels 8 days post-PSS exposure. (**A**) Graph showing 2-AG levels in the hippocampus 8 days post-PSS exposure. (**B**) Graph showing 2-AG levels in the hypothalamus 8 days post-PSS exposure. (**C**) Graph showing AEA levels in the hippocampus 8 days post-PSS exposure. (**D**) Graph showing AEA levels in the hypothalamus 8 days post-PSS exposure. Significant values are shown in the graphs.

There were significant differences in 2-AG levels among the groups in the hippocampus (Figure 5A) and hypothalamus (Figure 5B) areas [one-way ANOVA: F(3,56) = 15.0, *p* < 0.0001 and F(3,56) = 8.1, *p* < 0.00015, respectively]. Bonferroni post hoc tests confirmed significantly lower 2-AG levels in the hippocampus in the EBR group than in the unexposed, PBR, and MBR groups (*p* < 0.0001, *p* < 0.0001 and *p* < 0.0005, respectively). Moreover, in the hypothalamus, 2-AG levels were significantly lower in the EBR group than in the unexposed and PBR groups (*p* < 0.0001 and *p* < 0.0045, respectively). No significant differences were found in AEA between groups in the hypothalamic area, (Figure 5D). However, hippocampal AEA levels were significantly lower in the EBR group than in the unexposed and PBR groups (*p* < 0.0001 and *p* < 0.0001, respectively) (Figure 5C). These results indicate that, while in the acute aftermath, 2-AG levels rise in the group that will later develop extreme disruption in behavior; this disruption leads to a reduced neural expression of 2-AG.

## 3. Discussion

During a stressful event, there is a sub-sequential activation of the HPA-axis and rise in GC [41,42]. This rise activates a cascade of feedback inhibition processes terminating the acute activation of the HPA-axis [43,44,45]. Inadequate GC release, or insufficient activation of the HPA axis, shortly after traumatic or stressful events interferes with the processing and interpretation of stressful information, resulting in long-term disruptions of memory integration processes such as in the case of PTSD and PTSD-phenotype [33,46,47,48,49]. The cause of this insufficient HPA activation is still largely unknown. It was posited by our research team that this is mediated by an enhanced feedback inhibition of the HPA-axis [34], as supported by studies in humans [50,51,52]. However, one of the early components involved in the fast feedback inhibition of the HPA-axis in acute stress is the eCB 2-AG [5,10]. Our results demonstrate that only rats whose behavior was extremely disrupted (PTSD phenotype) in response to PSS displayed significantly higher CSF 2-AG levels 1–2 h after exposure, whereas animals whose behavior was less severely affected displayed no change in CSF 2-AG levels. Our findings showed an increased level of 2-AG in the CSF in the acute aftermath of stress (PSS exposure) in the PTSD phenotype group; this was negatively associated with urinary corticosterone secretions. These findings support three existing hypotheses: first, 2-AG is involved in the inhibition of GC immediately after stress; second, the inability to mount a GC stress response is indicative of a subsequent development of a PTSD-phenotype, as shown by the correlation between the hormonal stress response and the later assigned EBR group; third, the inability to mount a stress response and the subsequent disruption of behavior is mediated by an increased feedback inhibition, as supported by these findings.

Previous studies have shown that 2-AG is an early component in HPA feedback inhibition working in a retrograde manner in the hypothalamus, activated by GC membrane receptors [35,53,54,55]; together with our findings, this leads to the assumption that an over-activation of the feedback inhibition mechanism may be rooted either in the over activation of GC membrane receptors or the over-production of 2-AG brought on by the activation of these membrane receptors. While further studies are needed, this hypothesis might narrow the scope of inquiry. It is important to note that, while the action of GC in the paraventricular nucleus of hypothalamus, as well as in the amygdala, is mediated at least in part by 2-AG, it might not be the case in other brain regions, such as the hypothalamus, which are affected by GC during stress and play roles in the development of PTSD [36,54,56,57,58,59].

Eight days post-PSS, we found that rats exhibiting PTSD-phenotype displayed overall reduced levels of eCBs in the hypothalamus and hippocampus.

Our findings of reduced AEA levels in the hippocampus in the EBR group compared with the PBR group and unexposed controls are consistent with prior studies in rodents and humans, demonstrating a correlation between fear and other PTSD symptoms, as well as other psychiatric illnesses, with lower levels of AEA in the hippocampus [8,14,60,61,62], however, though a trend was apparent, we found no significant differences between rats whose behavior was extremely disrupted (i.e., PTSD-phenotype) and those exhibiting a minimal disruption in behavior.

Moreover, 2-AG levels were also decreased in the hippocampus compared with all other groups. While 2-AG is less studied in the context of PTSD symptomology, decreased levels of 2-AG in the hippocampus were also found to be related to PTSD symptoms such as anxiety, depression and memory disruption [8,63]; curiously, this was found to correlate with a reduced density of the target receptor for 2-AG (CB_1_) [64].

Our results showing reduced levels of 2-AG in the hypothalamus are intriguing. Current studies of 2-AG levels in the hypothalamus focus on its levels in reaction to stress and its regulation of the HPA-axis. The differences presented in this study might reflect further changes in HPA-axis regulation in the PTSD-phenotype group or a pre-existing condition leading to the initial disruption in response to a traumatic event, however further studies are needed. Furthermore, these differences were not significant between rats whose behavior was extremely disrupted and those exhibiting a minimal disruption in behavior.

Our study is not devoid of limitations, which should be addressed in future studies. First, measuring corticosterone or endocannabinoids levels at several time points following the exposure to stress may clarify the time course of alteration of these molecules and could shed light on the association between the HPA axis activity and the endocannabinoid system; the focus of an ongoing follow-up study. Second, one must take care not to be too literal in interpreting animal models; they should not be taken to comprehensively reflect the human disorder, but rather to approximate certain aspects of it. A third limitation of our study is that larger samples would have been preferable in the other experiments presented in this article, although the overall sample size was sufficient for statistical analyses.

Taken together, we propose that the eCB 2-AG is first involved in the development of behavioral disruption, brought on by the inadequate inhibition of the HPA-axis, mediated in part by an abnormal activation of the ECS. This disruption, in turn, reduces the neuronal expression of AEA and 2-AG in the hippocampus, contributing to PTSD symptomology [18,65,66,67].

## 4. Materials and Methods

All procedures were performed under strict compliance with ethical principles and guidelines of the NIH Guide for the Care and Use of Laboratory Animals. All treatment and testing procedures were approved by the Animal Care Committee of Ben-Gurion University of the Negev, Israel (IL-09-06-2014).

### 4.1. Animals

A sample of 62 adult male Sprague-Dawley rats, weighing 190–220 g on the day of surgery, was used. The rats were housed, 2–3 per cage, in a vivarium with a stable temperature, a 12:12 light-dark cycle (lights off at 07:00 pm; luminous emittance during the light phase: 200G50 lux), with unlimited access to food and water. All rats were maintained under this regime for a 1-week habituation period before the experiments began. All procedures were performed during the resting phase of the rats, between 07:30 and 18:30. During this period, corticosterone levels remained low and stable [68], but rats were more vulnerable to the traumatic stress exposure [69]. Thus, we assume that the circadian influence on corticosterone level and behavior did not interfere with the interpretation of our results.

### 4.2. Experimental Design

After a 7-day habituation period, rats (*n* = 62) were exposed to PSS/sham PSS for 15 min. CSF samples were collected 60–120 min after PSS to evaluate the levels of 2-AG and AEA. Urine corticosterone levels were also monitored 60–120 min following exposure. Following this procedure, animals were left undisturbed (2–3 per cage) for 7 days. On the 7th day post-PSS exposure, we conducted behavioral testing based on the CBC model [37,38,39,40]. These included elevated plus-maze (EPM) and acoustic startle response (ASR) tests. The behavioral data subsequently served for retrospective classification into behavioral response groups, as briefly detailed in (Figure S1). On day 8, rats were sacrificed, and their brains were collected for endocannabinoids. The experimental design used here is schematically depicted in Figure 1.

### 4.3. Predator Scent Stress (PSS)

Rats were individually placed on well-soiled cat litter, which was used by a cat for 2 days and sifted for stools. The rats were exposed to the litter for 10 min in a plastic cage (inescapable exposure) placed on a yard paving stone in a closed environment [37,38,39,40]. Sham-PSS (control) was administered under similar conditions, but the rats were exposed to fresh, unused cat litter.

### 4.4. Behavioral Measurements

The behavior of rats was assessed in the EPM and ASR paradigms, as described previously [37,38,39,40], and as briefly detailed in Supplementary Materials 1. All behavioral tests were video-recorded for future analysis using the ETHO-VISION program (Noldus Information Technology, Wageningen, The Netherlands), by an investigator blinded to the experimental protocol.

### 4.5. Cut-Off Behavioral Criteria (CBC) Model

The classification of individuals according to the degree to which their behavior was affected by a stressor is based on the premise that extremely compromised behavior in response to the priming trigger is not conducive to survival and is thus inadequate and maladaptive, representing a pathological degree of response. The severity of the response of each rat to the stressor is classified as either an extreme behavioral response (EBR), a partial behavioral response (PBR), or a minimal behavioral response (MBR) [37,38,39,40]. (See Figure S1).

### 4.6. Cerebrospinal Fluid (CSF) Collection Procedure

**Figure 6:**
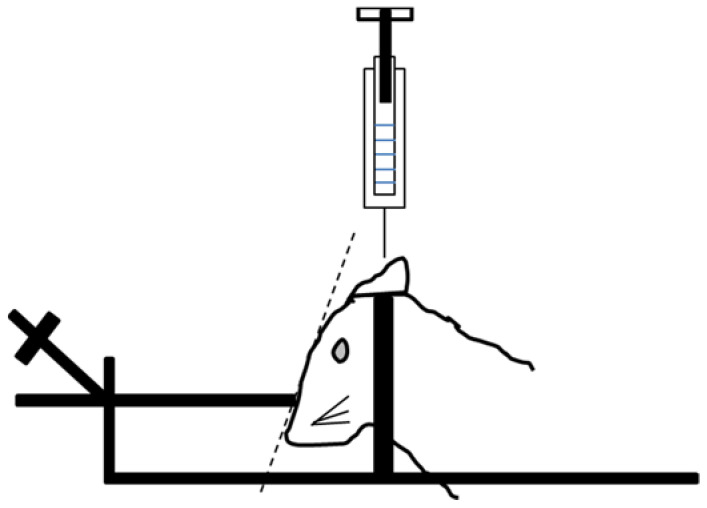
Fixation of rats in a stereotactic device during puncture of the great cerebral cistern. Adapted from ref. [70].

For CSF collection, the rats were anaesthetized with a combination of ketamine (70 mg/kg) and xylazine (6 mg/kg) i.p. and fixed in a stereotactic apparatus, with its body flexed downward, as described previously [70]. Briefly, the angle between the head of the rat and its body was set at 90°, as shown in Figure 6; punctured surfaces were positioned horizontally. The skin from the spinous process projecting at the initial part of the vertebral column and skull bones was cut along the median line (10–15 mm). The tissues were mechanically displaced from the midline of muscular fasciae on the neck until dura mater appeared. The dura mater looked like a stretched membrane and had a mat surface (rhombus). The parietal bone, vertebral column, and occipital protuberances formed the cerebral, caudal, and lateral corners of the rhombus, respectively. The membrane was perpendicularly punctured at the middle point of the midline (depth 1 mm). The maximum amount of the liquid was sampled. The needle was removed. A median longitudinal incision (1.5 mm) was made through the site of puncture in the dura mater. The residual liquid was removed from the cisterna magna (CM) with a cotton plug. The cavity of the CM was filled with freshly prepared acryl emulsion using a micropipette. After the induration of acryl, CM models were separated from surrounding tissues and examined in transmitted light. Profiles of the CM were visualized on the screen and sketched in the frontal and sagittal surfaces. The actual size of the CM was measured on models with a micrometer [71,72,73]. CSF sample volume ranged from 100–150 µL.

### 4.7. Urine Corticosterone Sampling

Urine samples were collected daily for corticosterone levels, by gently removing each rat to metabolic cages for 30 min, as described previously [74]. Animals were placed in these cages from their home cage and, once the procedure was complete, the animal was returned to its home cage. Rats were allowed to acclimate to the metabolic cage for 7 days before urine collection. Metabolic cages are regular cages with grooves along the floor, allowing for urine collection in suspended calibrated cylinders. All samples were immediately frozen (−80 °C) after collection. Samples were taken 60–90 min following exposure (between 12:30 and 14:00 h). Corticosterone concentrations were measured with a DSL-10-81000 ELISA kit according to the instructions of the manufacturer (Diagnostic Systems Laboratories, Webster, TX, USA) by a person blind to experimental procedures. All samples were measured in duplicate.

### 4.8. Analysis of Endocannabinoid Levels

The concentrations of the endocannabinoids, AEA and 2-AG, were measured in CSF, hypothalamus, and hippocampus tissues by LC-MS/MS, as described previously [75]. Briefly, rats were sacrificed by cervical dislocation and decapitation. Brains were partially thawed on ice and two brain regions were sampled: the hypothalamus and hippocampus. Each sample was weighed and transferred into individual vials for further homogenization. Tissue was homogenized in methanol (1 mL/100 mg of tissue), containing [2H4]-anandamide and [2H8]-2-AG as internal standards (Cayman Chemical, Ann Arbor, MI, USA). Lipids were extracted with chloroform (2 mL) and water (1 mL). The organic phases were dried under nitrogen (N2), reconstituted in chloroform (2 mL), and fractionated by open-bed silica gel column chromatography, as described [76]. The eluted fractions containing 2-AG and AEA were dried under nitrogen, and residues were suspended in chloroform/methanol (1/3, vol/vol; 60 μL). Analyses were conducted using a liquid chromatography/mass spectrometry (LC/MS) [76,77]. Moreover, 2-AG and AEA were separated on a ZORBAX Eclipse XDB-C18 column (Agilent Technologies, Santa Clara, CA, USA) using a linear gradient of methanol in water (from 85% to 90% methanol in 2.5 min), at a flow rate of 1 mL/min. To determine serum levels of 2-AG and AEA, lipids were eluted on a HP ODS Hypersil column (100 × 4.6 mm inner diameter, 5 µm particle size, Thermo Scientific, West Palm Beach, FL, USA), using a gradient of methanol in water (from 75% to 100% methanol in 35 min) at a flow rate of 0.5 mL/min. Column temperature was kept at 20 °C. MS detection was in the positive ionization mode, capillary voltage was set at 3000 V, and fragmentor voltage varied from 120 to 140 V. Nitrogen gas was used as a drying gas at a flow rate of 13 L per minute and a temperature of 350 °C. The nebulizer pressure was set at 30 p.s.i. Absolute amounts of 2-AG and AEA were quantified using a calibration curve [75].

### 4.9. Statistical Analyses

Data are presented as the mean ± SEM, unless otherwise specified. A *p* < 0.05 was considered to be statistically significant. For the behavioral results, the statistical analyses were performed using repeated measure (RM) analysis of variance (ANOVA). Bonferroni tests were used to examine differences between individual groups.

The behavioral data were transformed to percentage by using the CBC model: the prevalence of affected rats as a function of the rat group was tested by using cross-tabulation and nonparametric chi-squared tests. All nonparametric analyses were performed on raw data (and not on percentage).

## Data Availability

Not applicable.

## Supplementary Materials

Supplementary Materials can be found at https://www.mdpi.com/article/10.3390/ijms22126416/s1.

## Abbreviations

AEA	Anandamide
2-AG	2-arachidonoyl glycerol
ANOVA	Analysis of variance
CSF	Cerebrospinal fluid
ECS	Endocannabinoid system
EBR	Extreme behavioral response
EPM	Elevated plus-maze
GC	Glucocorticoid
HPA	Hypothalamus-pituitary-adrenal
MBR	Minimal behavioral response
PBR	Partial behavioral response
PSS	Predator scent stress
PTSD	Posttraumatic stress disorder
PVN	Paraventricular nucleus of the hypothalamus
