# Supplementary material for: Is PTSD-Phenotype Associated with HPA-Axis Sensitivity?: The Endocannabinoid System in Modulating Stress Response in Rats
Source: Int J Mol Sci. 2021 Jun 15;22(12):6416. doi: 10.3390/ijms22126416 (PMC8232809; doi:10.3390/ijms22126416)
Supplement: Supplementary file 1 [file ijms-22-06416-s001.zip › ijms-1217028-supplementary.pdf]

Supplementary Materials

1. The Cut-off Behavioral Criteria Model of PTSD

The behavioral responses of animals in both the elevated plus maze and acoustic startle response tests were first analyzed by group (e.g., blast-exposed vs. sham-exposed vs. unexposed). Subsequently, individual animals were classified according to their behavioral response pattern on both the elevated plus maze and acoustic startle response, by using the cut-off behavioral criteria model [37– 40], as exhibiting either "extreme behavioral response" (EBR) or "minimal behavioral response" (MBR). Those that fulfilled neither set of criteria were labeled exhibiting a "partial behavioral response" (PBR). This procedure is detailed in Figure S1.

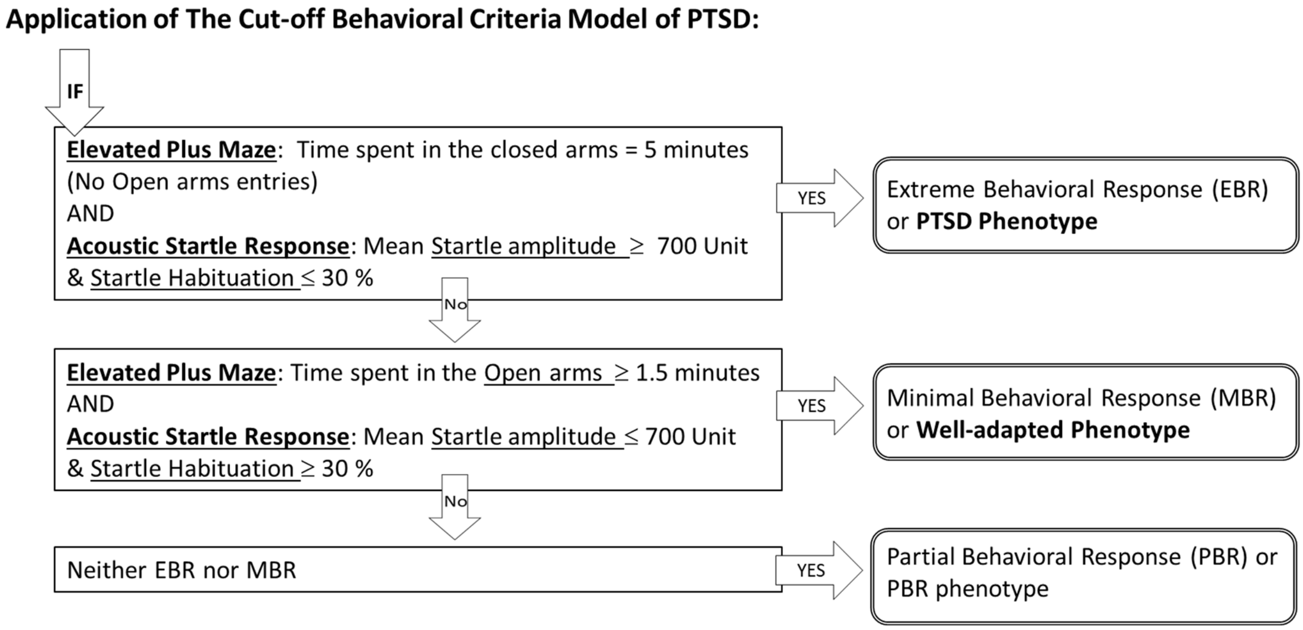

Figure S1. The cut-off behavioral criteria algorithm: To approximate the behavioral model to contemporary clinical conceptions of post-traumatic stress disorder (PTSD), we classified animals into groups according to degree of response to the stressor (PSS, predator scent stressor), i.e., the degree to which the behavior of an individual is altered or disrupted. To this end, behavioral criteria were defined and then complemented by the definition of cut-off criteria, which reflect the severity of response; this parallels inclusion and exclusion criteria applied in clinical research. Application of the cut-off behavioral criteria to the data: to maximize the resolution and minimize false positives, extreme responses to the stress in both the elevated plus-maze and acoustic startle response paradigms (performed sequentially) are required for "inclusion" into the extreme behavioral response (EBR) group. A negligible response in both paradigms is required for inclusion into the minimal behavioral response (MBR) group. Individuals that are not classified as having an EBR or an MBR are, by default, classified as having a partial behavioral response (PBR).

2. Behavioral Assessments

All rats underwent a number of different behavioral assessments. All behavioral tests were performed in a closed, quiet, light-controlled room between 10:00–16:00 h. All behavioral tests were video-recorded for future analysis using the ETHO-VISION program (Noldus Information Technology, Wageningen, The Netherlands), by an investigator blinded to the experimental protocol.

The elevated plus-maze: The maze is a plus-shaped platform with two opposing open and two opposing closed arms (surrounded by 41-cm high opaque walls on three sides) [74]. Rats were placed on the central platform facing an open arm and allowed one to explore the maze for 5 min. Each test was videotaped and subsequently scored by an independent observer. Arm entry was defined as entering an arm with all four paws. Be-

haviors assessed were: time spent (duration) in open and closed arms on the central platform; number of open and closed arm entries; and total exploration (entries into all arms). Total exploration was calculated as the number of entries into any arm of the maze, in order to distinguish between impaired exploratory behavior, exploration limited to closed arms (avoidance) and free exploration. "Anxiety Index", an index that integrates the elevated plus maze behavioral measures, was calculated. Anxiety Index values range from 0–1, where an increase in the index expresses increased anxiety-like behavior [23,139]

Acoustic startle response: Startle response was measured using two ventilated startle chambers (SR-LAB system, San Diego Instruments, San Diego, CA). The SR-LAB calibration unit was used routinely to ensure consistent stabilimeter sensitivity between test chambers and over time. Each Plexiglas cylinder rests on a platform inside a sound-proofed, ventilated chamber. Movement inside the tube is detected by a piezoelectric accelerometer below the frame. Sound levels within each test chamber are measured routinely using a sound level meter (Radio Shack) to ensure consistent presentation. Each test session started with a 5-min acclimatization period to background white noise of 68 dB, following by 30 acoustic startle trial stimuli in 6 blocks (110 dB white noise of 40 ms duration with 30 or 45 s inter-trial interval). Behavioral assessment consisted of: mean startle amplitude (averaged over all 30 trials) and percent of startle habituation to repeated presentation of the acoustic pulse. Percentage habituation—the percent change between the response to the first block of sound stimuli and the last—was calculated.
